# Identification of Potential Biomarkers and Related Transcription Factors in Peripheral Blood of Tuberculosis Patients

**DOI:** 10.3390/ijerph17196993

**Published:** 2020-09-24

**Authors:** Longxiang Xie, Xiaoyu Chao, Tieshan Teng, Qiming Li, Jianping Xie

**Affiliations:** 1 Cell Signal Transduction Laboratory, Bioinformatics Center, Department of Pathology, Institute of Biomedical Informatics, School of Basic Medical Sciences, Henan University, Kaifeng 475004, China; xielongxiang123@126.com (L.X.); 17334815782@126.com (X.C.); xiaoshan1220@163.com (T.T.); liqiming82@126.com (Q.L.); 2 State Key Laboratory Breeding Base of Eco-Environment and Bio-Resource of the Three Gorges Area, Key Laboratory of Eco-Environments in Three Gorges Reservoir Region, Institute of Modern Biopharmaceuticals, Ministry of Education, School of Life Sciences, Southwest University, Beibei, Chongqing 400715, China

**Keywords:** diagnostic biomarker, bioinformatics, PBMC, hub gene, tuberculosis

## Abstract

Tuberculosis (TB), one major threat to humans, can infect one third of the worldwide population, and cause more than one million deaths each year. This study aimed to identify the effective diagnosis and therapy biomarkers of TB. Hence, we analyzed two microarray datasets (GSE54992 and GSE62525) derived from the Gene Expression Omnibus (GEO) database to find the differentially expressed genes (DEGs) of peripheral blood mononuclear cell (PBMC) between TB patients and healthy specimens. Functional and pathway enrichment of the DEGs were analyzed by Metascape database. Protein-protein interaction (PPI) network among the DEGs were constructed by STRING databases and visualized in Cytoscape software. The related transcription factors regulatory network of the DEGs was also constructed. A total of 190 DEGs including 36 up-regulated genes and 154 down-regulated genes were obtained in TB samples. Gene functional enrichment analysis showed that these DEGs were enriched in T cell activation, chemotaxis, leukocyte activation involved in immune response, cytokine secretion, head development, etc. The top six hub genes (namely, LRRK2, FYN, GART, CCR7, CXCR5, and FASLG) and two significant modules were got from PPI network of DEGs. Vital transcriptional factors, such as FoxC1 and GATA2, were discovered with close interaction with these six hub DEGs. By systemic bioinformatic analysis, many DEGs associated with TB were screened, and these identified hub DEGs may be potential biomarkers for diagnosis and treatment of TB in the future.

## 1. Introduction

Tuberculosis (TB) usually caused by *Mycobacterium tuberculos* (Mtb) strains is a widespread and fatal infectious disease. It is considered that almost one third of the world’ s population is infected with this bacillus Mtb [1]. Approximately 9 million newly diagnosed TB patients and 1.5 million deaths occur every year. The obvious signs and symptoms of TB contain fever, night sweats, chills, appetite loss, weight loss, and fatigue [2], which lack specificity for auxiliary examination indices. So far, sputum smear microscopy and Mtb isolation in bacteriological culture are still the most traditional, classic, widely used tools for TB diagnosis [3]. But these tools need large amounts of time, and are not very accuracy [3], and the early diagnosis and effective treatment for TB patients is not easy to achieve. Therefore, discovering sensitive and specific biological markers of TB would not only provide new insights for the molecular and cellular processes involved in pathogenicity, but also establish a fast, sensitive and efficient way for diagnosis and treatment. Many studies have explored the potential biomarkers for the diagnosis and treatment of TB [4,5,6,7,8]. For instance, Sahin et al. suggested that a high molecular weight mucin-like glycoprotein CA-125, remarkably increased in active pulmonary TB compared to that in inactive TB or normal sample, may be a biomarker of pulmonary tuberculosis activity [9]. Suzuki et al. reported that macrophage activation marker *CD163* is found significantly higher in patients with active pulmonary TB than in control subjects, and its level is associated with mortality rate of pulmonary TB patients, indicating that *CD163* may be a potential diagnosis and prognosis biomarker in pulmonary TB [10]. Additionally, serum interleukin (IL)-6 level was increased in active pulmonary tuberculosis compared with normal healthy subjects, and may function in immuneprotection of the host against Mtb infection [11]. In 2014, Zhuang et al. found that circular RNA Hsa_circ_0005836 was downregulated in the PBMCs of active pulmonary tuberculosis compared with health controls by using high-throughput sequencing and qRT-PCRs (real-time quantitative reverse transcription-polymerase chain reactions), and it may be treated as a novel diagnosis marker and potential therapeutic target of active pulmonary tuberculosis [4]. Recently, Fu et al. showed that several dysregulated circRNAs including circRNA_103017 and circRNA_059914 have potential diagnostic value for TB, and circRNA_101128-let-7a interplay may function in PBMCs response to Mtb infection [12]. Although several factors have been discovered, the efficiency and accuracy of TB diagnosis is still unsatisfactory [13]. Therefore, it is very necessary to find new potential diagnosis and therapeutic biomarkers for TB patients.

With the development of genomics technology, there are large amounts of data in the field of TB research [14,15]. Bioinformatics can be used to mine the existing data at relatively low cost to discover novel diagnosis biomarkers of TB. In this study, we screened the DEGs between PBMC of TB patients and healthy donors in two individual GEO datasets, and subsequently performed the gene ontology (GO), pathway enrichment and PPI network analysis.

## 2. Material and Methods

**Figure 1:**
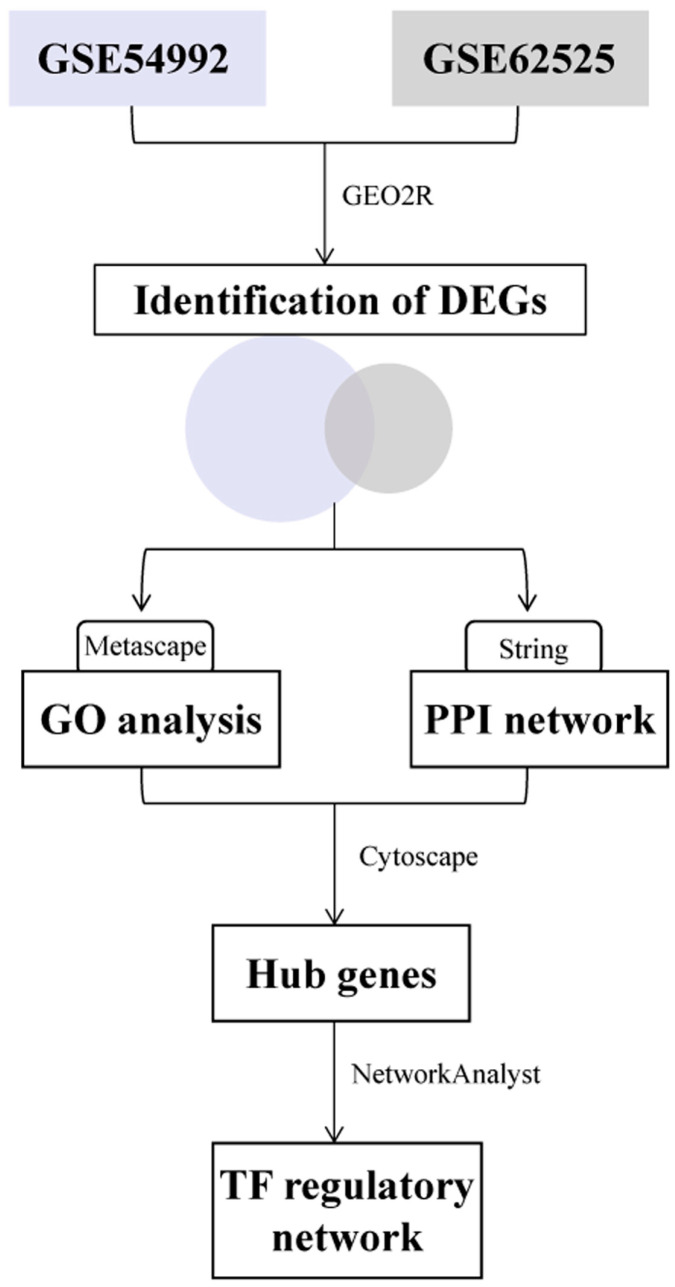
The flow diagram of this study.

The flow diagram for the entire process is shown in Figure 1. Analysis of gene expression data was performed using web tools and software described below.

### 2.1. Microarray Data

Two gene expression datasets including GSE54992 [16] and GSE62525 [15] were downloaded from Gene Expression Omnibus (GEO) of NCBI. The platform of GSE54992 was GPL570 “HG-U133_Plus_2” Affymetrix Human Genome U133 Plus 2.0 Array platform. The platform of GSE62525 was GPL16951 Phalanx Human OneArray platform. GSE54992 comprise of 15 peripheral blood samples from nine TB cases and six normal specimens. GSE62525 contained 28 peripheral blood samples from seven TB cases and seven normal specimens (every individual has two replicate samples). Patients derived from GSE54992 were from the Shenzhen Third People’s Hospital, China. Diagnosis of active TB was relied on patients’ clinical symptoms, chest radiography, microscopy for AFB (acid fast bacilli), sputum Mtb culture and response to anti-TB chemotherapy. These 15 PBMC samples were collected before anti-tuberculosis treatment [16]. Patients derived from GSE62525 were admitted to the the Institutional Review Board of Taoyuan General Hospital, Ministry of Health and Welfare, Taoyuan, Taiwan. Diagnosis of each subject was according to the sputum smear test, T-SPOT TB test, and a chest radiograph. These 14 PBMC samples were collected before anti-tuberculosis treatment [15].

### 2.2. Data Processing

The interactive web tool GEO2R was used to analyze the gene expression data of microarrays and find DEGs [17]. Only genes with |logFC| > 1 and *p*-values < 0.05 were selected as DEGs. Subsequently, Venny 2.0) was performed to obtain the overlapping components in the both GSE54992 and GSE62525.

### 2.3. GO and KEGG Pathway Enrichment Analysis

Metascape web-server was used for Gene ontology (GO) analysis and Kyoto Encyclopedia of Genes and Genomes (KEGG) pathway enrichment analysis [18]. We set the Terms or pathways with *p*-value < 0.01, min overlap genes = 3, and min enrichment factor > 1. as the cutoff criteria.

### 2.4. Protein–Protein Interaction (PPI) Network Construction and Module Analysis

The functional protein–protein interaction (PPI) analysis is important for interpreting the potential molecular mechanisms of key cellular activities in pathogenicity. Search Tool for the Retrieval of Interacting Genes (STRING) database (http://string-db.org) [19] was used to construct PPI network of high expression genes and low expression genes, respectively. Interaction score of 0.4 was treated as the cut-off criterion and Cytoscape (http://www.cytoscape.org/) was used to visualize the PPI network [20]. Hub genes were selected with connection degree > 9. Then, the Molecular Complex Detection (MCODE) in Cytoscape software was performed to obtain he modules within PPI network with MCODE score > 2 and number of nodes > 4. The functional enrichment analysis of the genes in each module was also completed by Metascape.

### 2.5. TFs Regulatory Network of Hub Genes

NetworkAnalyst web tool was used to find the transcriptional factors (TFs) of the hub genes and construct the gene-TF regulatory network [21]. NetworkAnalyst is a web-based tool that contains systemic profiling, meta-analysis and interpretation of gene expression data [21].

## 3. Results

### 3.1. Identification of DEGs in PBMC from TB Patients

**Figure 2:**
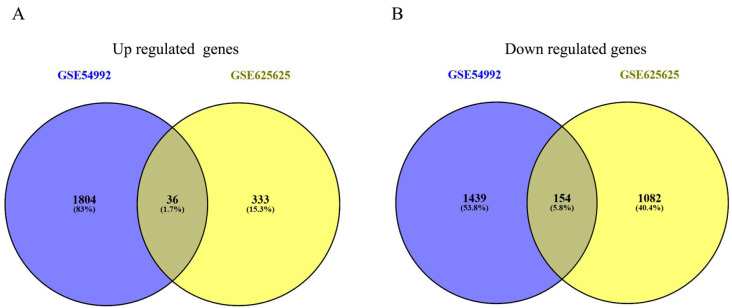
Selection of 190 commonly DEGs from the GSE54992 and GSE62525. Different color areas showed different datasets. The cross areas meant the overlapping changed DEGs. (**A**) Up regulated genes. (**B**) Down regulated genes.

Two microarray datasets including GSE54992 and GSE62525 were uploaded to the GEO2R to screen DEGs in PBMC between normal specimens and TB patients. Finally, 3433 DEGs were obtained in GSE54992 dataset, which include 1840 up-regulated genes and 1593 down-regulated ones. 1890 DEGs were obtained in GSE62525 dataset, which include 369 up-regulated genes and 1521 down-regulated ones. A total of overlapped 190 genes (36 up-regulated and 154 down-regulated) were found in this two datasets (Figure 2A,B). The detailed results are also shown in Table S1. These 190 overlapping genes were treated as candidate DEGs and used for further analysis.

### 3.2. GO Analysis of DEGs

**Figure 3:**
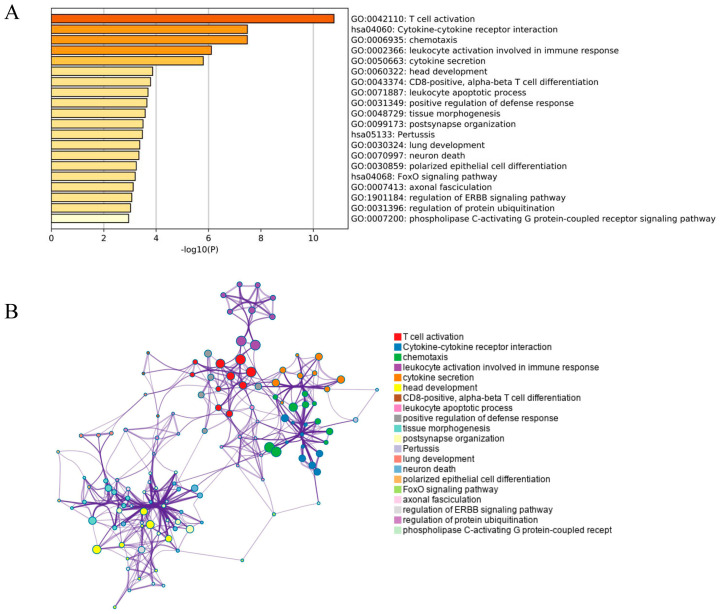
GO and KEGG pathway enrichment analysis of DEGs. (**A**) GO terms and KEGG pathway were presented. (**B**) Network of the enriched terms and pathways.

The GO functions including molecular function, biological process and cellular component, and KEGG pathway enrichment analysis of 190 DEGs were performed in the Metascape. As shown in Figure 3A, 17 terms and three pathways were found to be involved in the DEG enrichment analysis, and these DEGs were mainly enriched T cell activation, chemotaxis, leukocyte activation involved in immune response, cytokine secretion, CD8-positive, α-/β-T cell differentiation, leukocyte apoptotic process, etc. In addition, these enriched terms were tightly associated with each other and clustered into intact networks (Figure 3B). KEGG pathway enrichment analysis showed that three significantly enriched pathway including cytokine-cytokine receptor interaction, pertussis, and FoxO signalling pathway (Figure 3A). To find some differential pathways in the TB patients compared to the Healthy doors, we also performed KEGG pathway enrichment analysis of 36 up-regulated genes and 154 down-regulated genes in Metascape, respectively. The results showed that 36 up-regulated genes were mainly enriched in cytokine-cytokine receptor interaction, and 154 down-regulated genes were also mainly enriched in cytokine-cytokine receptor interaction and FoxO signaling pathway (Figure S1).

### 3.3. PPI Network Construction and Hub Gene Selection

**Figure 4:**
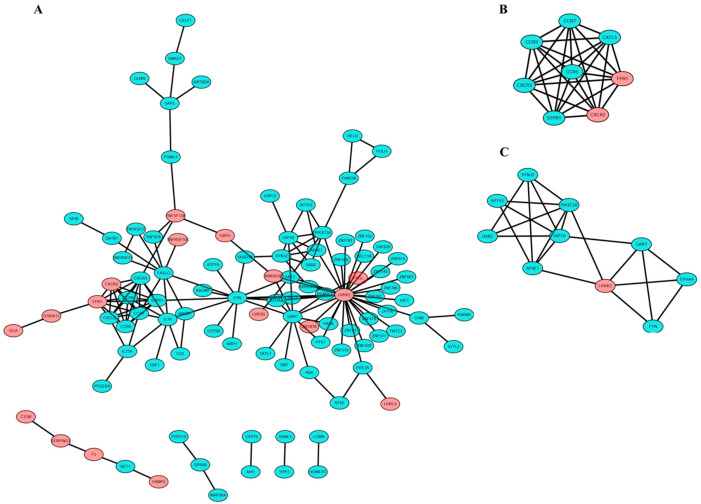
PPI network and top two modules of 190 DEGs ((**A**) PPI network of DEGs; (**B**,**C**) top module 1–2).

**Figure 5:**
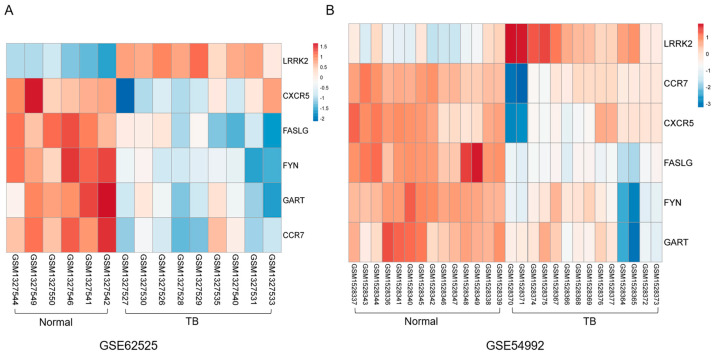
Heat map of top 6 hub genes’ expression in both (**A**) GSE62525 and (**B**) GSE54992. Blue represents downregulation, and red represents upregulation.

PPI network analysis has been treated as a useful tool for exploring the biological responses in health and disease. According to the STRING database and Cytoscape software, 188 in the 190 commonly changed DEG were filtered into the DEGs PPI network complex, including 188 nodes and 149 edges (Figure 4). Hub genes are a series of genes that play an vital role in the diverse biological process. In the relevant pathways, the regulation of other genes is often regulated by these hub genes. The top six genes with highest interaction degrees were identified in this study, including LRRK2 (leucine rich repeat kinase 2), FYN (Src family tyrosine kinase), GART (phosphoribosylglycinamide formyltransferase), CCR7 (C-C motif chemokine receptor 7), CXCR5 (chemokine receptor type 5), and FASLG (Fas ligand). The LRRK2 gene had the highest degree, suggesting its potentially critical role in the pathogenicity. The heatmap of these six hub genes’ expression from GSE62525 and GSE54992 was shown in Figure 5. 

Receiver operating characteristic (ROC) analysis was the common-used method for binary assessment, and then it was used to assess the effectiveness of the transcriptional expression of six hub genes to discriminate TB patients from healthy control. The computed area under the curve (AUC) value ranging from 0.5 to 1.0 shows the discrimination ability from 50 to 100%. The results showed that these genes have the strong ability to discriminate TB from healthy control with AUC ≥ 0.9 and *p* < 0.0001 (data not shown). Six such genes are considered as candidates for novel TB biomarkers.

### 3.4. Module Analysis

To understand the potential function of genes in modules at the molecular level, we used MCODE plugin to analyze the module of the PPI network, and chose the most significant two modules for further analyses according to the degree of importance. The PPI network of DEGs is illustrated in Figure 5A and top two modules are shown in Figure 5B. 

**Figure 6:**
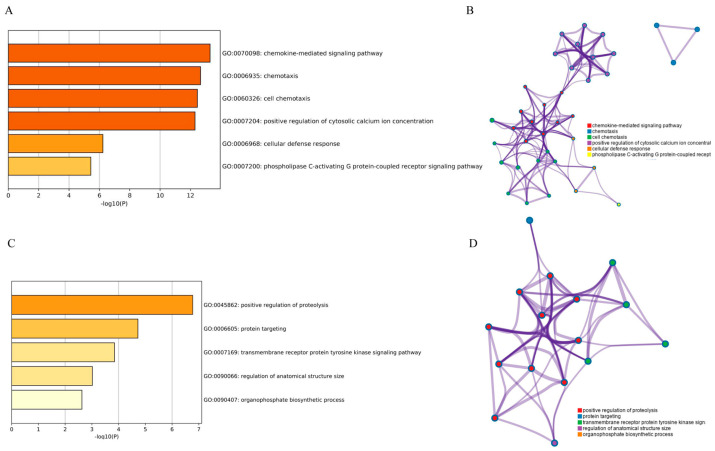
GO and KEGG pathway enrichment analysis of Module 1 (**A**,**B**) and Module 2 (**C**,**D**).

Functional enrichment analysis demonstrated that Module 1 was remarkably enriched in chemokine-mediated signaling pathway, chemotaxis, cell chemotaxis, positive regulation of cytosolic calcium ion concentration and G-protein coupled receptor signaling pathway (Figure 6A,B), while Module 2 was mainly related to positive regulation of proteolysis, protein targeting, transmembrane receptor protein tyrosine kinase signaling pathway, etc. (Figure 6C,D).

### 3.5. TF Regulatory Network Analysis of 6 Hub Genes

**Figure 7:**
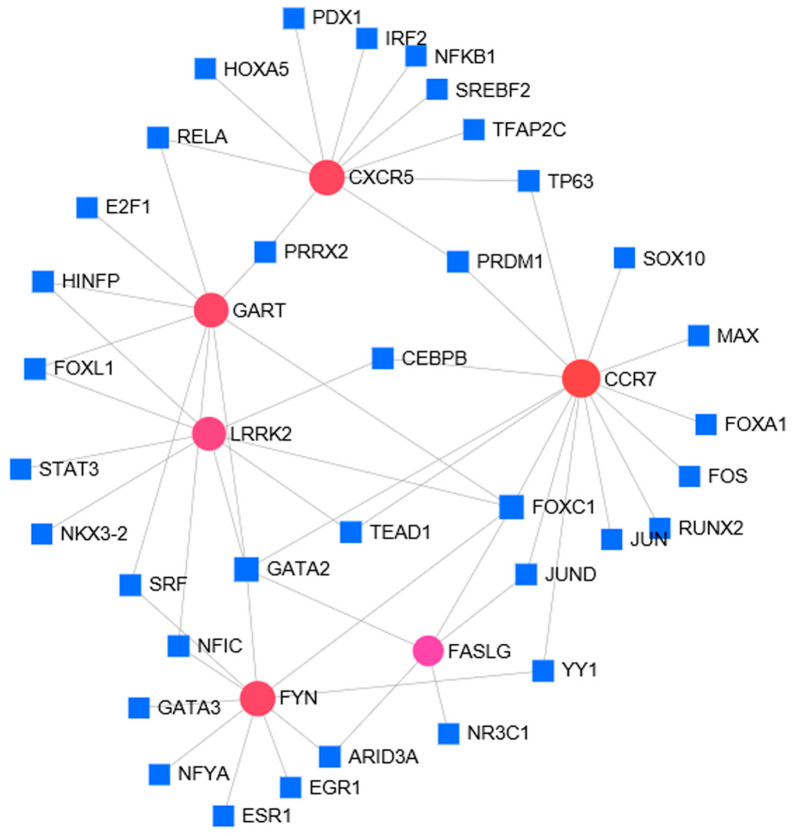
The hub gene-TF regulatory network. Red circle means the hub gene and blue square means the transcription factor.

For these six identified hub genes, we constructed a gene-TF regulatory network including 56 interaction pairs among six genes and 41 TFs (Figure 7). CCR7 was found to be regulated by 14 TFs, FYN was regulated by 10 TFs, FASLG was regulated by five TFs, CXCR5 was regulated by 10 TFs, GART was regulated by nine TFs, LRRK2 was regulated by eight TFs. In addition, several TFs were found to regulate more than one hub gene, and 15 TFs were found with a connectivity degree ≥ 2 in the gene-TF regulatory network, which shows that these TFs may have close interactions with these hub DEGs (Table 1).

For instance, FOXC1 was predicted to regulate LRRK2, FYN, GART, CCR7, CXCR5, and FASLG; GATA2 was found to regulate FASLG, GART, LRRK2, CCR7.

## 4. Discussion

Early and accurate diagnosis of TB and quick treatment are very important for preventing its spread [22]. Here, we used several bioinformatics tools to analyze the differential gene expression between patients’ PBMC and healthy persons’ PBMC, to provide novel view for unraveling pathogenesis of TB. According to the criterion for DEGs (adjusted *p*-value < 0.05 and |logFC| > 1), we finally got 190 DEGs including 36 up-regulated genes and 154 down-regulated genes. Then we used the Metascape database for GO enrichment analysis and KEGG pathway enrichment analysis. Based on GO analysis results, the majority of the DEGs were involved in molecular function, specifically protein binding rather than in biological process or cellular component (Figure 3). GO function analysis found that more significant enrichments in the DEGs were T cell activation, chemotaxis, leukocyte activation involved in immune response, cytokine secretion, head development, etc. Wang et al. found that differentially expressed proteins and among multidrug-resistance tuberculosis (MDR-TB) patients, drug-sensitive tuberculosis (DS-TB) patients, and healthy controls could construct a network mainly in complement and coagulation cascades, providing potential biomarkers for MDR-TB diagnosis [23]. Zhang et al. demonstrated that a proline deletion in IFNAR1 inhibits IFN-signaling and is related with decreased susceptibility to TB in humans, indicating a potential function for the IFNAR1 inter-domain region in cytokine-cytokine receptor interaction and signal transduction [24]. Notably, different platforms that contains different number of probes may affect the number of overlapping genes. Meanwhile, if more TB patients’ samples or database included, the overlapping genes may be less. For example, we found another gene expression dataset GSE65517 from NCBI GEO. The platform of GSE65517 was GPL10558 Illumina HumanHT-12 V4.0 expression beadchip. GSE65517 comprise of 6 peripheral blood samples from three TB cases and three normal specimens. We used GEO2R to analyze the DEGs of GSE65517 (|logFC| > 1 and *p* < 0.05), and finally found 2502 DEGs (1188 up-regulated genes and 1314 down-regulated genes). Among 190 overlapping DEGs from both GSE54992 and GSE62525, 43 genes were found in 2502 DEGs from GSE65517 (Figure S2). The reason may be due to the limited number (only three TB cases and three normal specimens) of TB samples in GSE65517 dataset. Due to this limited number of samples, this dataset was not included in this study.

By establishing a PPI, six key genes were found. *LRRK2* (leucine-rich repeat kinase 2) has been demonstrated that its mutations are associated with Crohn’s disease, Parkinson’s disease, ulcerative colitis, and mycobacterial infections. In the GSE57275, we found that when compared with Mtb un-infected lung, *LRRK2* was significantly up-regulated in the Mtb infected lung (*p* < 0.0001) (Figure S3) [25]. Recently, Härtlova et al. found that *LRRK2* is a negative regulator of *M. tuberculosis* phagosome maturation in macrophages [26]. *LRRK2*-dependent cellular pathway can regulate *M. tuberculosis* replication in macrophages by regulating phagosome maturation [26]. In the GSE20050, we found that when compared with normal lung parenchyma, *LRRK2* was significantly down-regulated in caseous tuberculosis granulomas (*p* = 0.0361), but *CCR7* and *FYN* was upregulated (*p* = 0.0024 and *p* = 0.0486, respectively) (Figure S4) [27]. Khader and colleagues demonstrated that *CD4*+*CXCR5*+ T cells have a protective function in the immune response against TB infection [28]. In Mtb-infected lungs, activated *CD4*+*CXCR5*+ T cells were found to be accumulated and can produce proinflammatory cytokines. In addition, *CXCR5* gene deficient mice with defective T cell localization in lung parenchyma were likely to become infected TB. *CXCR5* expression in T cells play an important role in accurate T cell localization within TB granulomas, reinforced macrophage activation, protected against Mtb infection, and promoted lymphoid follicle formation [28]. The Fas/Fas-ligand (FasL) system is involved in regulating apoptosis and the immune response and can be used by mycobacteria to evade the immune response. Mustafa et al. found that the level of soluble FasL was significantly higher in children than that in adults, and FasL may function in immune modulation and pathogenesis of TB [29]. Lu et al. found that *CD83* and *CCR7* are highly expressed in myeloid dendritic cell (DC) from active pulmonary tuberculosis patients, but low expressed in plasmacytoid DC [30].

TFs, the main regulators of gene expression, are related with the pathogenic processes of TB. In this study, we also found some TFs with close interactions with hub DEGs. Forkhead box C1 (FOXC1) is a transcription factor that is essential for mesenchymal lineage specification and organ development during normal embryogenesis [31]. In addition, mutations of FOXC1 can lead to the heritable Axenfeld-Rieger Syndrome and other congenital disorders. Twelve mutations in GATA2 were found to be correlated with the autosomal dominant and sporadic monocytopenia and mycobacterial infection (MonoMAC) syndrome [32]. Our study showed that these TFs formed a connected regulatory network with hub DEGs, thus implied that the dynamic changes in these TF activities may play critical roles in controlling the expression and function of hub DEGs associated with pathogenic processes of TB.

The bioinformatic analysis performed in this study concentrated on two datasets with a total of 13 normal samples and 16 TB patient samples. As a result, the sample size was limited, and high-quality studies with larger sample sizes would be included to support this study in future study. In addition, the number of the DEGs we identified (190 DEGs) was too many, but whether all of them could be biomarkers is subject to expirical confirmation. The gene profiles may differ between early and late TB. We checked the clincial information of GSE54992 and GSE62525, but there were no detailed information for whether these samples are early TB. We tried to find the the early stage TB patients’ samples in GEO database. Unfortunately we could not obtain new related datasets. We shall maintain our focus on the updated datasets about early stage of TB.

## 5. Conclusions

In this study, we analyzed the datasets of RNA profiles of PBMC from TB patients by the bioinformatic tools and obtained 190 common DEGs between the two datasets. Then we performed a PPI network and network modules analysis of these DEGs, and six Hub genes were screened. Key TFs, such as FOXC1 and GATA2 were also found with tight interactions with these six hub DEGs from the gene-TF network. Collectively, these results may provide a basis for the screening of drug candidates and diagnosis biomarkers for TB. However, further studies are required to reveal the functions of the DEGs in physiological and pathological processes through cell and animal experiments. It would be also valuable to follow up this DEG identification analysis by correlating it to treatment outcomes in patients in the dataset used by the authors as a means of zeroing on biomarkers that have the most impact on the patient.

## Figures and Tables

**Table 1:** The potential TFs of hub genes.

TFs	Genes	Count
FOXC1	LRRK2, FYN, GART, CCR7, CXCR5, and FASLG	6
GATA2	FASLG, GART, LRRK2, CCR7	4
PRDM1	CXCR5, CCR7	2
TP63	CXCR5, CCR7	2
PRRX2	CXCR5, GART	2
RELA	CXCR5, GART	2
YY1	FYN, CCR7	2
NFIC	FYN, GART	2
SRF	FYN, GART	2
CEBPB	LRRK2, CCR7	2
TEAD1	LRRK2, CCR7	2
JUND	FASLG, CCR7	2
FOXL1	LRRK2, GART	2
HINFP	LRRK2, GART	2
ARID3A	FYN, FASLG	2

## Supplementary Materials

The following are available online at https://www.mdpi.com/1660-4601/17/19/6993/s1, Figure S1. GO and KEGG pathway enrichment analysis of DEGs. A, GO terms and KEGG pathway of up-regulated genes were presented; B, GO terms and KEGG pathway of down-regulated genes were presented. Figure S2. Selection of 43 commonly DEGs between the GSE65517 and overlapping DEGs. Different color areas showed different datasets. The cross areas meant the overlapping changed DEGs. Figure S3. LRRK2 was significantly up-regulated in the Mtb infected lung compared with normal tissues. Figure S4. CCR7 and FYN was upregulated in GSE20050. Table S1. The list of 190 DEGs in both GEO datasets.
