# Supplementary figures and images for: Identification of Potential Biomarkers and Related Transcription Factors in Peripheral Blood of Tuberculosis Patients
Source: Int J Environ Res Public Health. 2020 Sep 24;17(19):6993. doi: 10.3390/ijerph17196993 (PMC7579196; doi:10.3390/ijerph17196993)

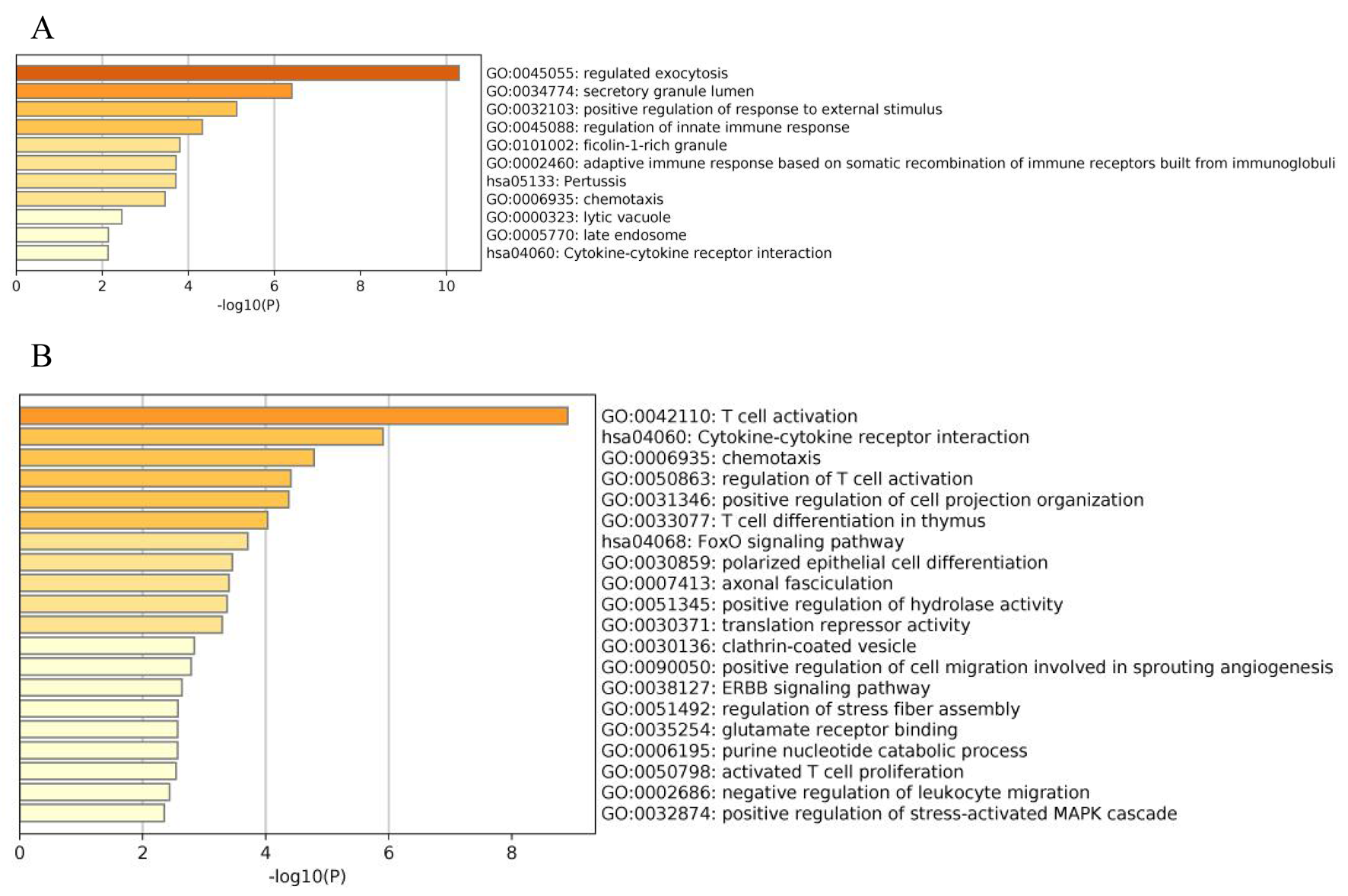

Supplement: Supplementary file 1 [file ijerph-17-06993-s001.zip › Figure S1.tif]

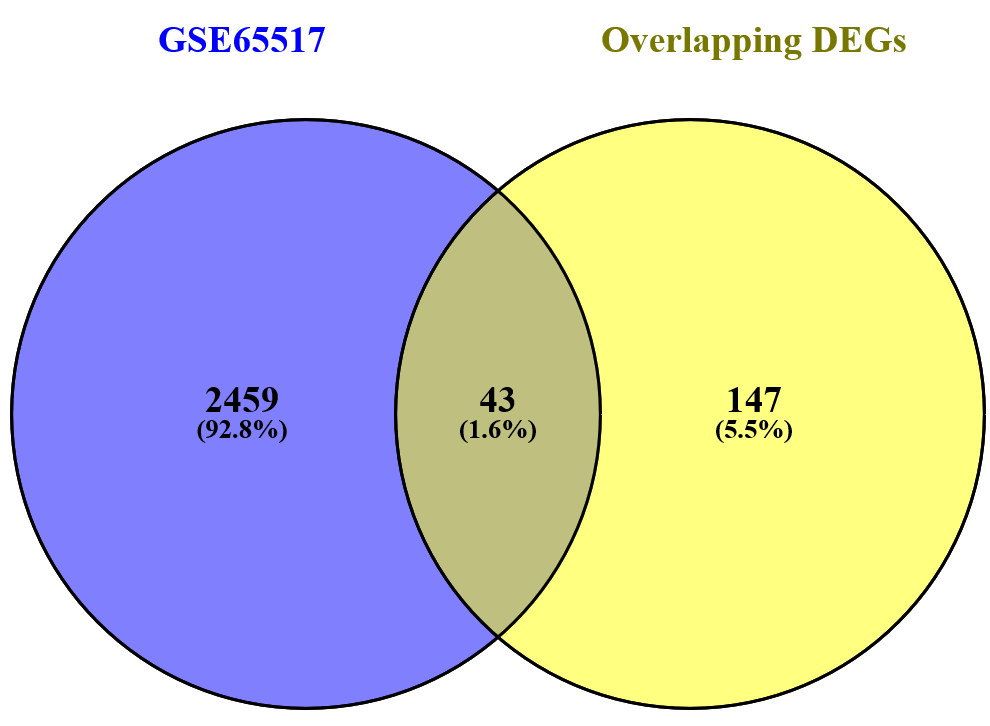

Supplement: Supplementary file 1 [file ijerph-17-06993-s001.zip › Figure S2.tif]

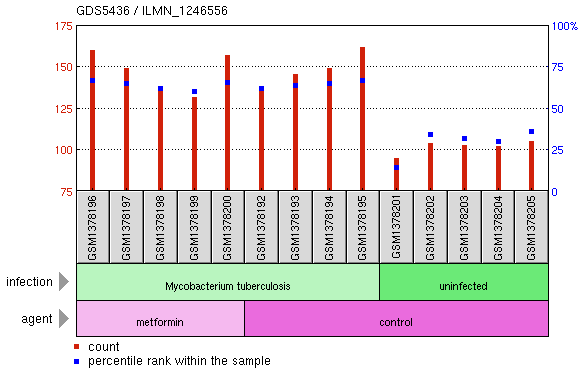

Supplement: Supplementary file 1 [file ijerph-17-06993-s001.zip › Figure S3.png]

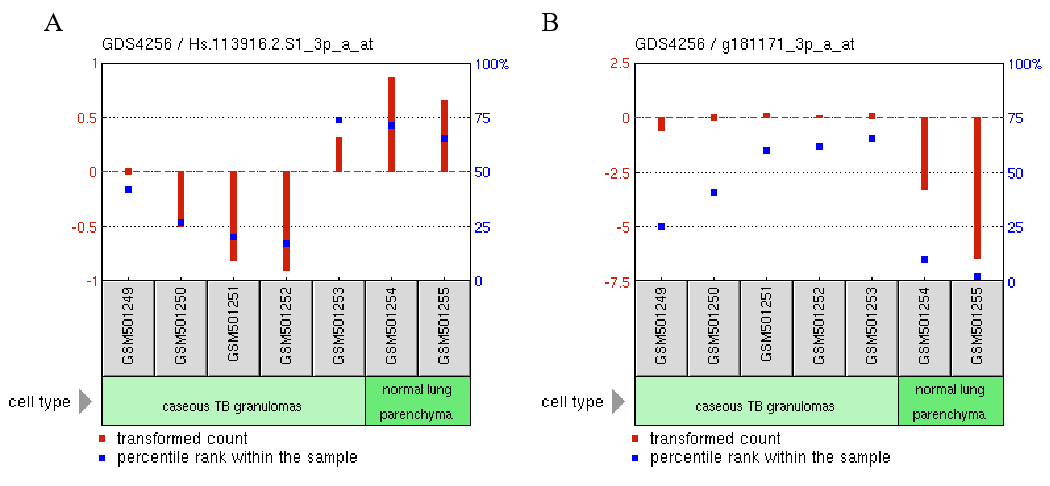

Supplement: Supplementary file 1 [file ijerph-17-06993-s001.zip › Figure S4.jpg]
